# Differences in symptoms and problems experienced by patients with a life-threatening disease in specialized palliative care and basic palliative care—a nationwide cross-sectional study

**DOI:** 10.1007/s00520-025-10071-1

**Published:** 2025-11-05

**Authors:** Maiken Bang Hansen, Mogens Groenvold, Mette Raunkiær, Tina Broby Mikkelsen, Leslye Rojas-Concha, Cecilie Lindström Egholm

**Affiliations:** 1 Palliative Care Research Unit, Department of Geriatrics and Palliative Medicine GP, Copenhagen University Hospital - Bispebjerg and Frederiksberg, University of Copenhagen, Copenhagen, Denmark; 2 Department of Cancer and Cancer Screening, The Danish Health Care Systems Quality Institute, Copenhagen, Denmark; 3 Department of Public Health, University of Copenhagen, Copenhagen, Denmark; 4 The Danish Knowledge Centre for Rehabilitation and Palliative Care, REHPA, University of Southern Denmark, Odense University Hospital, Odense, Denmark

**Keywords:** Palliative care, Symptom assessment, Needs assessment, Quality of life, Cardiovascular diseases, Neurological diseases, Lung diseases, Kidney diseases, Cancer

## Abstract

**Background:**

Patients with a life-threatening disease often experience palliative care needs (symptoms and problems) before death, and those with the most complex needs should be those who get access to specialized palliative care. To examine if that is the case, studies are needed comparing palliative care needs of patients with life-threatening cancer and non-cancer diseases admitted to specialized palliative care to patients receiving basic palliative care.

**Aims:**

To compare palliative care needs among patients with a life-threatening disease admitted to specialized palliative care and patients receiving basic palliative care.

**Design:**

A secondary data analysis of symptoms/problems reported by patients in basic palliative care or at admittance to specialized palliative care in Denmark. Ordinal logistic regression was performed to study differences in the probability of experiencing each symptom/problem among patients receiving specialized palliative care vs. basic palliative care, controlled for possible confounders.

**Setting/participants:**

Patients with a life-threatening disease who completed the EORTC QLQ-C15-PAL questionnaire.

**Results:**

The study included 6367 patients. The odds of experiencing symptoms, impaired physical functioning, impaired emotional functioning, and poor quality of life were higher at admittance to specialized palliative care compared to an ongoing basic palliative care, especially impaired physical functioning (OR 8.3, 95% CI 6.6–10.5) and fatigue (OR 5.1, 95% CI 4.1–6.5).

**Conclusions:**

Patients in specialized palliative care had higher levels of symptoms and problems than patients with a life-threatening disease receiving basic palliative care, especially non-cancer patients. Future research should study changes in symptoms/problems during the disease trajectory among patients with life-threatening diseases to improve referrals to specialized palliative care.

## Introduction

Patients with a life-threatening disease may receive palliative care to relieve their symptoms and problems [1]. It may be general/basic palliative care provided by professionals who do not have palliative care as their main function or specialized palliative care provided by professionals with palliative care as their main function [2, 3].

In high-income countries, it is estimated that around 75% would benefit from palliative care before they die [4, 5]. Due to limited capacity, not all patients in need of specialized palliative care receive it, and this problem is expected to increase in the future due to aging populations [6–10]. Nationwide data from different countries show that only 17–19% receive specialized palliative care before they die in Denmark and Sweden [7, 11], whereas 46% receive specialized palliative care in the last year of life in Australia [9]. Patients with a non-cancer disease are less likely to receive specialized palliative care than cancer patients [6–10].

Because the capacity of specialized palliative care is limited, it is very important to provide high-quality basic palliative care for patients with life-threatening diseases and that the patients who get referred to specialized palliative care are those with the most complex palliative care needs (high levels of and/or several symptoms/problems). To identify palliative care needs and to improve the quality of palliative care, the European Association for Palliative Care (EAPC) has recommended the use of validated patient-reported outcome (PRO) tools [12, 13]. PRO tools are important to use to obtain a comprehensive needs assessment, as clinicians tend to underestimate the frequency [14, 15] and severity [16–18] of their patients’ symptoms and problems.

We have not been able to identify any studies looking at how palliative care needs differ for patients with life-threatening diseases who do and do not receive specialized palliative care in the same country or region. Therefore, the aim of this study was to compare palliative care needs (symptoms, problems, and quality of life) among patients with life-threatening diseases admitted to specialized palliative care and patients receiving basic palliative care.

## Methods

### Study design

This study was a secondary data analysis of cross-sectional symptom/problem data reported by patients with life-threatening diseases in basic palliative care and specialized palliative care, respectively, in a 1-year period (November 1, 2021, to October 31, 2022). The collection of PRO data is elaborated later in the “Methods” section.

### Setting

In Denmark, specialized palliative care is provided by 42 palliative care teams in hospitals and by hospices [7]. Fifteen are hospital-based palliative care teams providing palliative care in their own hospital department, delivering specialized consultations in other hospital departments and providing home-based palliative care; eight additional teams also have inpatients; 15 hospices only have inpatients, whereas the remaining four hospices have inpatients and provide home-based palliative care. Basic (also called generalist) palliative care is provided in the rest of the healthcare system. Compared to non-cancer patients, cancer patients are more often referred (65% vs. 4%) and more often receive (50% vs. 2%) specialized palliative care before their death in Denmark [19].

### PRO-data at admittance to specialized palliative care

In Denmark, it is mandatory for all specialized palliative care services to report information on patients referred to them to the Danish Palliative Care Database [20]. The database includes sociodemographic and clinical information and symptoms and problems reported by patients who completed the European Organisation for Research and Treatment of Cancer Quality of Life Questionnaire Core 15 Palliative Care (EORTC QLQ-C15-PAL). Patients whom the healthcare professionals believe are physically and cognitively able to complete the EORTC QLQ-C15-PAL questionnaire are asked to do so at admittance to specialized palliative care (i.e., 1–3 days prior to the start date of specialized palliative care or at the start date of specialized palliative care).

The EORTC QLQ-C15-PAL is a shortened version of the EORTC QLQ-C30 adapted to cancer patients in palliative care [21], but has also been used among non-cancer patients in specialized palliative care in Denmark since 2010 [20]. EORTC QLQ-C15-PAL has been validated in several studies in patients with advanced cancer [21–28]. The EORTC QLQ-C15-PAL questionnaire assesses the severity of nine symptoms/problems by two multi-item function scales (physical and emotional functioning), two multi-item symptom scales (fatigue and pain), and five single-item symptom scales (dyspnea, insomnia, appetite loss, constipation, and nausea) together with a single item on overall quality of life. Patients answer to which degree they have experienced each symptom and problem on a 4-point scale from “not at all” to “very much.” A 7-point scale is used to assess overall quality of life, where 1 corresponds to “very poor” and 7 to “excellent.” All questions refer to the past week, except for physical functioning, where no time frame is specified.

### PRO-data from basic palliative care

In 2019, the Danish Health Data Authority established a national Clinical Coordination Group, which included healthcare professionals from different specialties from hospital departments and professionals from municipalities. With input from patients, the group developed a PRO-tool with the main purpose of identifying palliative care needs in the healthcare system outside specialized palliative care, i.e., in basic palliative care [29, 30]. The development of the new tool (the “PRO-Pall”) was completed in 2021 [31].

This study included the PRO-data collected in a feasibility test of PRO-PALL among adult patients with chronic and/or progressive life-threatening cancer or lung, kidney, or heart diseases. The PRO-data were collected in three municipalities, a research clinic, and in eight hospital departments: one cardiology department, three pulmonary medicine departments, one nephrology department, one oncology department, and two surgical departments [32]. The sites volunteered to participate in the feasibility test, and the PRO-PALL was incorporated into usual care processes.

The first part of the PRO-PALL is the EORTC QLQ-C15-PAL [21, 31]. PRO-PALL also includes two items on physical symptoms and six items on social and existential problems as well as WISP (Write In three Symptoms/Problems), allowing the patient to add up to three additional symptoms/problems using open-ended questions [32, 33]. In this study, only answers on the 15 EORTC QLQ-C15-PAL items were included since the purpose was to compare them with symptoms and problems reported at the start of specialized palliative care, where only the EORTC QLQ-C15-PAL is available.

### Population

Inclusion criteria were as follows:

- 18 + years of age
- Life-threatening cancer or lung, heart, or kidney diseases
- Physically and cognitively able to complete the EORTC-QLQ-C15-PAL/PRO-Pall
- Completed the EORTC QLQ-C15-PAL at admission to specialized palliative care or the PRO-PALL questionnaire while receiving care or treatment from the municipality or at a hospital department not specialized in palliative care

### Variables

Outcomes in the studies were overall quality of life and nine symptoms/problems (pain, dyspnea, sleeplessness, appetite loss, constipation, nausea, fatigue, emotional functioning, physical functioning).

The exposure variable was the setting, i.e., basic compared to specialized palliative care.

Covariates controlled for in the ordinal regression analyses were diagnosis (cancer, heart disease, lung disease, kidney disease), sex, and age.

### Statistics

The 15 EORTC QLQ-C15-PAL items were converted into ten 0–100 scales according to the scoring manual [21, 34]. Higher function scale scores represent better physical or emotional functioning, or better quality of life, whereas higher symptom scale scores represent worse symptoms. Symptom/problem mean scores were computed.

The ten symptom/problem/quality of life scales were dichotomized into a new variable for experiencing a symptom/problem at least “a little” (symptom scores ≥ 33, functional scores ≤ 67) or not (symptom score < 33, function score > 67). The prevalence of each symptom/problem was calculated as the proportion of patients experiencing the symptom/problem according to setting (specialized vs. basic palliative care) and diagnosis (cancer vs. non-cancer).

Ordinal logistic regression analyses were performed to study the associations between setting (specialized vs. basic palliative care) and each outcome while controlling for the effect of diagnosis, sex, and age. If the patient had EORTC data from admittance to more than one palliative care service, only data from the first admittance was included.

The analyses were performed using SAS Enterprise Guide 8.3.

## Results

### Characteristics of the study population

**Fig. 1:**
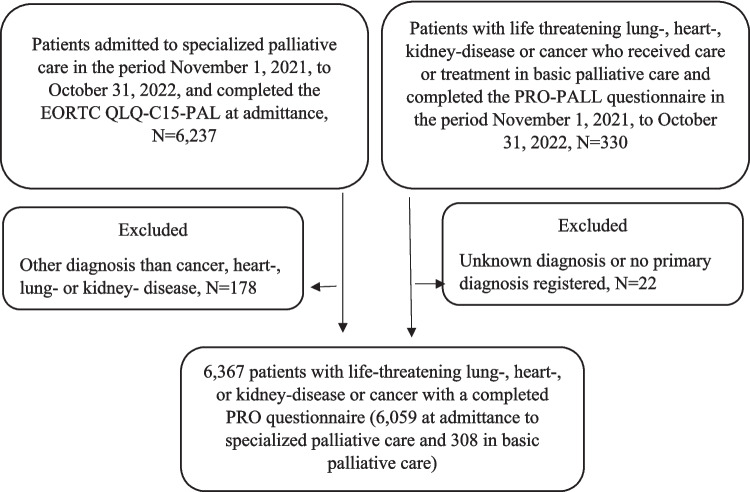
Flowchart of patients included in the study

This study included 308 patients from 12 sites providing basic palliative care and 6059 patients admitted to specialized palliative care (Fig. 1).

The study included 49.1% women with a median age of 72 years (Table 1). The median age and gender distribution were similar between settings, whereas the distribution of diagnostic groups differed, with a higher proportion of cancer patients in specialized palliative care (Table 1).

**Table 1:** Characteristics of the study population

Total, ***N*** (%)	All		Basic palliative care		Specialized palliative care	
	6367	100	308	100	6059	100
Sex						
Female, *N* (%)	3129	49.1	152	49.3	2977	49.1
Male, *N* (%)	3238	50.9	156	50.7	3082	50.9
Age						
Range (*N*, years)	6367	18–102	308	31–97	6059	18–102
Median (*N*, years)	6367	72	308	72	6059	72
Diagnosis						
Cancer, *N* (%)	5817	91.4	112	36.4	5705	94.2
Cardiovascular disease, *N* (%)	122	1.9	41	13.3	81	1.3
Respiratory disease, *N* (%)	367	5.8	130	42.2	237	3.9
Kidney disease, *N* (%)	61	1.0	25	8.1	36	0.6
Patients reporting the highest level (“very much”) of symptoms and functioning problems on PRO-PALL						
Pain items						
Had pain, *N* (%)	1764	27.7	51	16.6	1713	28.3
Pain interfered with daily activities, *N* (%)	1638	25.7	39	12.7	1599	26.4
Short of breath (dyspnea), *N* (%)	1214	19.1	52	16.9	1162	19.2
Trouble sleeping (sleeplessness), *N* (%)	970	15.2	33	10.7	937	15.5
Lacked appetite (appetite loss), *N* (%)	2037	32.0	38	12.3	1999	33.0
Been constipated (constipation), *N* (%)	769	12.1	20	6.5	749	12.4
Felt nauseated (nausea/vomiting), *N* (%)	686	10.8	14	4.5	672	11.1
Fatigue items						
Felt weak, *N* (%)	2678	42.1	49	15.9	2629	43.4
Were tired, *N* (%)	3097	48.6	77	25.0	3020	49.8
Emotional functioning items						
Felt tense, *N* (%)	880	13.8	18	5.8	862	14.2
Felt depressed, *N* (%)	534	8.4	23	7.5	511	8.4
Physical functioning items						
Trouble taking short walk outside of the house, *N* (%)	3026	47.5	69	22.4	2957	48.8
Need to stay in bed or a chair during the day, *N* (%)	3053	48.0	64	20.8	2989	49.3
Need help with eating, dressing, washing yourself or using the toilet, *N* (%)	1275	20.0	11	3.6	1264	20.9

### Mean symptom/problem and quality of life scores

**Fig. 2:**
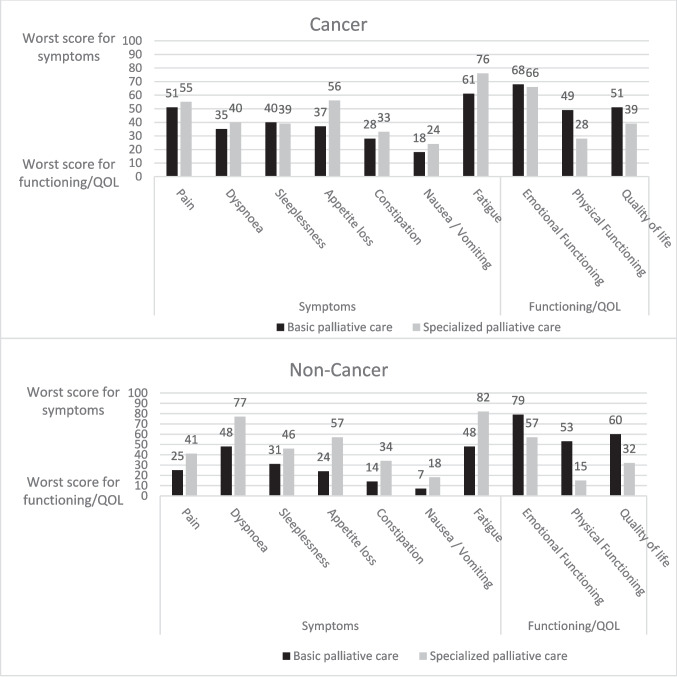
Mean symptom, functioning and overall quality of life scores according to setting and for cancer compared to non-cancer patients. The worst symptom score is 100, whereas the worst functioning and quality of life score is 0

Worse levels of symptoms, functioning, and quality of life were found for patients in specialized palliative care compared to patients in basic palliative care, especially among non-cancer patients (Fig. 2). Only sleeplessness among cancer patients had a slightly better (lower) score in specialized palliative care.

### Proportions experiencing symptoms and problems

**Fig. 3:**
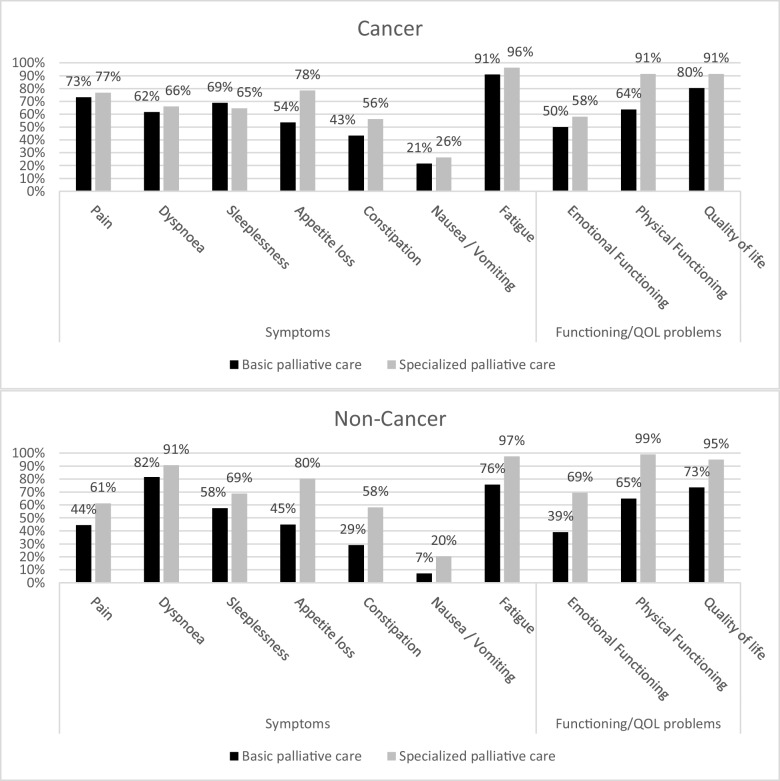
Proportions of patients experiencing symptoms and problems at least “a little” according to setting and for cancer and non-cancer patients

More than 50% of cancer and non-cancer patients experienced fatigue, dyspnea, sleeplessness, impaired physical functioning, and poor quality of life at least a little in basic and specialized palliative care, respectively (Fig. 3). Except for sleeplessness among cancer patients, the prevalence of all symptoms/problems was higher in specialized palliative care compared to basic palliative care (Fig. 3).

### Regression analyses

Patients in specialized palliative care had significantly higher odds of experiencing symptoms and problems compared to patients in basic palliative care (Table 2), especially higher odds of experiencing poor physical functioning (OR 8.3, 95% CI 6.6–10.5), fatigue (OR 5.1, 95% CI 4.1–6.5), and poor quality of life (OR 4.3, 95% CI 3.4–5.4). Gender, age, and diagnosis were in many cases significantly associated with the odds of experiencing symptoms/problems (Table 2). Diagnoses were strongly associated with dyspnea and the odds of dyspnea were higher among patients with lung disease (OR 9.9, 95% CI 7.8–12.5) and heart disease (OR 2.4, 95% CI 1.7–3.4), respectively, compared to cancer patients.

**Table 2:** Odds ratios and 95% CI for higher levels of symptoms, lower functioning and lower QOL, respectively, for patients in specialized palliative care compared to patients outside specialized palliative care from ordinal logistic regression

	Pain (*N* = 6331)			Dyspnea (*N* = 6287)			Sleeplessness (*N* = 6261)			Appetite (*N* = 6267)			Constipation (*N* = 6242)		
	OR	95% CI		OR	95% CI		OR	95% CI		OR	95% CI		OR	95% CI	
Specialized palliative care (yes vs. no)	**1.8**	**1.4**	**2.2**	**2.6**	**2.0**	**3.3**	**1.4**	**1.1**	**1.8**	**3.7**	**2.9**	**4.7**	**2.3**	**1.8**	**3.0**
Age (10-year increase)	**0.9**	**0.9**	**0.9**	**1.1**	**1.1**	**1.2**	**0.8**	**0.8**	**0.9**	**1.2**	**1.2**	**1.3**	**1.0**	**1.0**	**1.1**
Men (vs. women)	**0.9**	**0.8**	**0.9**	1.0	0.9	1.1	**1.2**	**1.1**	**1.3**	1.0	0.9	1.1	1.1	1.0	1.2
Diagnosis															
Heart (vs. cancer)	**0.3**	**0.2**	**0.4**	**2.4**	**1.7**	**3.4**	1.2	0.9	1.7	0.8	0.6	1.2	0.7	0.5	1.1
Respiratory (vs. cancer)	**0.4**	**0.4**	**0.5**	**9.9**	**7.8**	**12.5**	**1.3**	**1.0**	**1.6**	0.9	0.7	1.1	1.0	0.8	1.2
Renal (vs. cancer)	0.8	0.5	1.3	0.7	0.4	1.1	1.1	0.7	1.8	1.2	0.7	1.9	1.0	0.6	1.6
	Fatigue (*N* = 6180)			Nausea (*N* = 6271)			Poor emotional (*N* = 6020)			Poor physical (*N* = 6216)			Poor QOL (*N* = 5635)		
	OR	95% CI		OR	95% CI		OR	95% CI		OR	95% CI		OR	95% CI	
Specialized palliative care (yes vs. no)	**5.1**	**4.1**	**6.5**	**2.3**	**1.7**	**2.9**	**2.3**	**1.8**	**2.9**	**8.3**	**6.6**	**10.5**	**4.3**	**3.4**	**5.4**
Age (10-year increase)	**1.2**	**1.1**	**1.2**	**0.9**	**0.9**	**1.0**	**0.9**	**0.9**	**1.0**	**1.3**	**1.2**	**1.3**	**1.0**	**1.0**	**1.1**
Gender (men vs. women)	**0.8**	**0.8**	**0.9**	**0.7**	**0.6**	**0.8**	**0.9**	**0.8**	**0.9**	**0.9**	**0.8**	**0.9**	1.0	0.9	1.1
Diagnosis				^**a**^			^**a**^			^**a**^			^**a**^		
Heart (vs. cancer)	1.0	0.7	1.5	0.8	0.6	1.2	1.0	0.7	1.4	1.3	1.0	1.9	0.9	0.6	1.3
Respiratory (vs. cancer)	**1.3**	**1.1**	**1.6**	**0.6**	**0.5**	**0.8**	**1.9**	**1.5**	**2.3**	**2.1**	**1.7**	**2.6**	**1.5**	**1.2**	**1.9**
Renal (vs. cancer)	1.0	0.6	1.6	1.0	0.6	1.7	0.7	0.5	1.2	1.4	0.9	2.2	1.3	0.8	2.1

Significant (*P* < 0.05) odds ratios in bold

^a^Overall significant association between diagnosis and outcome

Odds ratios > 1 indicate more severe symptoms, lower (worse) functioning, or lower quality of life compared to the reference group

## Discussion

### Main findings

In basic and specialized palliative care, more than half of cancer and non-cancer patients experienced fatigue, dyspnea, sleeplessness, impaired physical functioning, and poor quality of life. Patients in specialized palliative care had significantly higher odds of experiencing symptoms and problems compared to patients in basic palliative care. Differences in the risk of symptoms/problems between settings (basic vs. specialized palliative care) were more pronounced among non-cancer patients than cancer patients.

### What this study adds

Using nationwide patient-reported symptoms and problems, this study found that patients with life-threatening diseases admitted to specialized palliative care had higher odds of symptoms/problems compared to patients in basic palliative care, especially for impaired physical functioning. This is expected since patients with the most complex symptoms/problems should be those who get access to specialized palliative care. Also, patients admitted to specialized palliative care in Denmark are often admitted close to death (median survival time is 39 days after admittance) [7]; and as death approaches, the risk of symptoms and problems increases. This has been documented in systematic reviews including cancer patients with an expected survival of a maximum of 3 months, where dyspnea, appetite loss, fatigue, and poor physical function were associated with shorter survival time [35–37]. Among cancer patients referred to specialized palliative care, studies have also found impaired physical function/wellbeing [38–51], problems related to eating/weight loss [38, 39, 41–48, 50–57], dyspnea [38, 41, 42, 44–49, 51, 54, 55, 57–60], and fatigue [38, 39, 42–44, 48, 51, 53, 56] to be associated with shorter survival time. In heart failure patients, a study from the USA found that among patients who were followed in the last 6 months of their life, functional impairments, median depression scores, and the proportion reporting severe pain or dyspnea increased as death approached [61].

In our study, the difference in the prevalence of symptoms/problems between basic and specialized palliative care was larger among non-cancer patients than among cancer patients. Previous studies comparing symptoms and problems among cancer and non-cancer patients with life-threatening diseases outside specialized palliative care have also found nausea/vomiting [62, 63], constipation [62, 64], and emotional problems (sadness/depression and anxiety) [62, 64, 65] to be more prevalent among cancer patients. We found dyspnea to be more prevalent among non-cancer patients in basic palliative care, which was also found in previous studies comparing cancer patients to non-cancer patients overall [62], patients with COPD [63, 65, 66] and kidney disease [64], respectively. Fatigue and impaired physical function were experienced by the majority of patients in basic palliative care in our study. Previous studies also found that fatigue/lack of energy and impaired physical function were frequently experienced by both cancer and non-cancer patients outside specialized palliative care [62–65, 67].

In our study, non-cancer patients often had similar or higher risk of symptoms/problems in specialized palliative care compared to cancer patients. The reason for this may be that non-cancer patients are less likely to be referred to specialized palliative care before death in Denmark compared to cancer patients, and therefore, perhaps only the highly complex non-cancer patients are referred [19].

In our study, more than half of the patients in specialized palliative care experienced each of the measured symptoms/problems, except for nausea/vomiting. Symptom/problem prevalence in specialized palliative care has varied across previous studies among cancer and non-cancer patients, respectively, but many studies have also found a high prevalence of pain, fatigue, appetite loss/anorexia, and dyspnea, respectively [44, 68–88].

We found dyspnea, fatigue, and impaired physical functioning among more than half of the patients in basic palliative care. In previous studies including cancer and/or non-cancer patients with life-threatening diseases who did not receive specialized palliative care, some symptoms and problems were also experienced frequently: at least 40% experienced fatigue [62–65, 67, 89–97], drowsiness [62, 65, 67, 90], cough [62, 64, 65, 67, 89–92], and worries [62, 64, 90, 94], respectively. The dyspnea prevalence has also often been at least 50% [64, 65, 67, 89–92, 95], although it was reported to be lower in some studies [61, 66, 94, 96, 97]. Most previous studies also found physical functioning problems among a minimum of 50% of the included patients [63, 89, 94, 97] but not all [96].

Across studies (including our study), there are large variations in the proportion of patients experiencing each symptom/problem in basic as well as specialized palliative care. The variation might be due to differences in the selection of patients (e.g., diagnosis), in the assessment tools and thresholds used, and in the capacity of and the referral criteria for specialized palliative care.

Patients with heart disease and especially respiratory disease in this study had significantly higher odds of dyspnea compared to cancer patients. Similarly, previous studies outside specialized palliative care found cancer patients to have a lower risk of dyspnea compared to non-cancer patients overall [62], and to patients with chronic obstructive pulmonary disease (COPD) [63, 65, 66] or heart failure [63]. Studies among patients referred to specialized palliative care have also found non-cancer patients had a higher risk of dyspnea compared to cancer patients [98–101], and two systematic reviews found that more than 70% of patients with advanced COPD receiving palliative care often experienced dyspnea [102, 103].

The fact that many patients in basic palliative care in this study had symptoms and problems identified when using the PRO-PALL questionnaire underlines the need for healthcare professionals outside specialized palliative care to be adequately trained/educated in providing palliative care, including being trained in using patient-reported outcome tools to identify palliative care needs. It is highly important that palliative education/training is available to healthcare professionals since oncologists and other healthcare professionals today have insufficient education in palliative care in many countries [104, 105]. Systematic symptom assessment in services providing basic palliative care may also help to identify and refer patients with the most complex palliative care needs early in the disease trajectory to specialized palliative care services [105, 106].

### Strengths and limitations

The main strength in this study is that it included nationwide patient-reported symptoms/problems from basic and specialized palliative care services during a 1-year period, which enables comparison of patients’ symptomatology across settings. It is also a strength that the study includes cancer and non-cancer patients. Another strength is that all the need assessments (PRO questionnaires) completed at the start of palliative care in one of the 42 Danish specialized palliative care services during the study period were included in the study and that 12 sites providing basic palliative care (eight hospital departments, one research department, and three municipalities) were included. Overall, we believe the internal validity is high, although some limitations exist. First, convenience sampling was used to include patients from the 12 sites providing basic palliative care; thus, the patients are not necessarily representative for all Danish patients with life-threatening diseases outside specialized palliative care. Only in one of the sites providing basic palliative care was data collected on the proportion completing the PRO questionnaire, and in that hospital department, it was 75% (99 of 132 patients). The proportion of patients completing the PRO questionnaire at the start of specialized palliative care was 67%.

Some of the differences found, in this study, in symptomatology between patients in basic and specialized palliative care may be explained by differences in the proportion of inpatients and outpatients in the two settings as well as differences in prognosis. In specialized palliative care, 31% were inpatients and 69% were outpatients. We do not have the exact proportion of inpatients and outpatients in basic palliative care, but we know 39% were from outpatient services and 25% from inpatient services, whereas the remaining 36% were from services with a mix of inpatients and outpatients. Therefore, the proportion of outpatients could be from 39 to 75% (39% + 36%). It is also likely that the prognosis was better in basic palliative care compared to specialized palliative care; but unfortunately, information was not available at the individual level for patients in basic palliative care regarding in/outpatient status and prognosis. Another limitation is that EORTC QLQ-C15-PAL was developed for cancer patients, but the questionnaire does, however, seem to assess the most prevalent symptoms and problems experienced by cancer and non-cancer patients in palliative care [107, 108]. In addition, the EORTC QLQ-C15-PAL questions plus eight extra questions (included in the PRO-PALL questionnaire) were found easy to understand and relevant according to most of the 270 patients with cancer as well as other life-threatening illnesses [109]. Different factors may affect the generalizability of the findings in this study, such as the organization of palliative care in different countries.

## Conclusions

Patients with life-threatening diseases admitted to specialized palliative care had a higher risk of symptoms and problems than patients receiving basic palliative care.

Future studies are needed to better understand changes in the level of symptoms/problems during the disease trajectory among patients with life-threatening diseases. This might help improve referral to specialized palliative care, especially for non-cancer patients.

## Data Availability

No datasets were generated or analysed during the current study.
